# Research on Drought Stress Detection in the Seedling Stage of Yunnan Large-Leaf Tea Plants Based on Biomimetic Vision and Chlorophyll Fluorescence Imaging Technology

**DOI:** 10.3390/biomimetics11010056

**Published:** 2026-01-08

**Authors:** Baijuan Wang, Weihao Liu, Xiaoxue Guo, Jihong Zhou, Xiujuan Deng, Shihao Zhang, Yuefei Wang

**Affiliations:** 1 College of Agricultural & Biotechnology, Zhejiang University, Hangzhou 310013, China; wangbaijuan2023@163.com (B.W.); zhoujihong@zju.edu.cn (J.Z.); 2 College of Tea Science, Yunnan Agricultural University, Kunming 650201, China; liuweihao2210@163.com (W.L.); 18488871225@163.com (X.G.); 2021066@ynau.edu.cn (X.D.); 3 Mechanical & Electrical Engineering College, Wuhan Donghu College, Wuhan 430071, China

**Keywords:** drought stress detection, YOLOv13, multi-scale linear attention, bio-inspired computing

## Abstract

To address the issue of drought level confusion in the detection of drought stress during the seedling stage of the Yunnan large-leaf tea variety using the traditional YOLOv13 network, this study proposes an improved version of the network, MC-YOLOv13-L, based on animal vision. With the compound eye’s parallel sampling mechanism at its core, Compound-Eye Apposition Concatenation optimization is applied in both the training and inference stages. Simulating the environmental information acquisition and integration mechanism of primates’ “multi-scale parallelism—global modulation—long-range integration,” multi-scale linear attention is used to optimize the network. Simulating the retinal wide-field lateral inhibition and cortical selective convergence mechanisms, CMUNeXt is used to optimize the network’s backbone. To further improve the localization accuracy of drought stress detection and accelerate model convergence, a dynamic attention process simulating peripheral search, saccadic focus, and central fovea refinement in primates is used. Inner-IoU is applied for targeted improvement of the loss function. The testing results from the drought stress dataset (324 original images, 4212 images after data augmentation) indicate that, in the training set, the Box Loss, Cls Loss, and DFL Loss of the MC-YOLOv13-L network decreased by 5.08%, 3.13%, and 4.85%, respectively, compared to the YOLOv13 network. In the validation set, these losses decreased by 2.82%, 7.32%, and 3.51%, respectively. On the whole, the improved MC-YOLOv13-L improves the accuracy, recall rate and mAP@50 by 4.64%, 6.93% and 4.2%, respectively, on the basis of only sacrificing 0.63 FPS. External validation results from the Laobanzhang base in Xishuangbanna, Yunnan Province, indicate that the MC-YOLOv13-L network can quickly and accurately capture the drought stress response of tea plants under mild drought conditions. This lays a solid foundation for the intelligence-driven development of the tea production sector and, to some extent, promotes the application of bio-inspired computing in complex ecosystems.

## 1. Introduction

As an important component of the unique agricultural industry on the Yunnan Plateau, the tea industry plays a crucial role in the province’s economic development [1]. According to statistics from the Agricultural Information Center of the Yunnan Provincial Department of Agriculture and Rural Affairs in 2015, the cultivation area of Yunnan large-leaf tea trees, as one of the core genetic resources of the tea industry in Yunnan, accounts for more than 90% of the total tea tree area in the province. In recent years, the combined effect of abnormal temporal and spatial distribution of precipitation and the increasing frequency of extreme high temperatures has led to frequent natural disasters [2]. Among these, the increasing frequency and severity of drought events have significantly disrupted the critical growth stages of Yunnan large-leaf tea trees, resulting in notable adverse impacts on their yield and quality [3].

Traditional drought monitoring of tea trees primarily relies on soil moisture content measurement, leaf relative water content and leaf water potential, stomatal conductance and transpiration rate, osmotic adjustment-related indicators and electrolyte leakage rate, as well as manual visual symptom assessment and regular field inspections [4,5,6,7]. However, these indicators are often responsive signals, which typically show significant changes only after stress has caused irreversible structural or functional damage, making them difficult to meet the early warning requirements. Additionally, the operation and maintenance costs of field sensor nodes are high, and their power supply reliability and weather resistance are limited. Therefore, efficiently and accurately monitoring drought stress in Yunnan large-leaf tea trees has become one of the key issues limiting the improvement of intelligent management levels in tea plantations [8].

In recent years, with the deep integration of artificial intelligence and agricultural science, deep learning technologies have been widely applied to plant phenotype recognition, feature extraction, and data processing [9]. Algorithms based on image recognition, pattern classification, and deep learning can mine key features from vast amounts of plant phenotype data, enabling efficient perception and intelligent analysis of drought stress signals [10]. The integration of biomimetic vision and deep learning, moreover, provides a new technological approach for intelligent monitoring of drought stress [11].

Lei Zhou and colleagues addressed the issue of drought grading in poplar seedlings being confounded by varietal differences in cross-variety contexts [12], and proposed a multi-task deep learning model capable of simultaneously predicting both variety and drought level. During the construction phase, the model performs instance segmentation on leaves, petioles, and leaf veins, and combines automatically synthesized images with their annotations to significantly reduce annotation costs. In addition, two targets are simultaneously learned in a multi-task framework that shares features and uses joint loss to alleviate domain offset. The results show that the multi-task MobileNet model has a variety recognition accuracy of 99.07% and a drought classification accuracy of 75.93% on the independent test set, which is more than 5 percentage points higher than the single-task basic model. This study proves to a certain extent that joint supervision helps the model to learn robust features related to variety and stress at the same time, thus improving the accuracy and stability of grading detection.

Yue Hu et al. proposed a bionic visual detection method for the pain points that are difficult to be accurately detected by crop aphids in complex backgrounds [13]. Inspired by the mechanism that human eyes preferentially gaze at highly discriminative local structures in complex scenes. The network labels the aphid head separately during the construction of the data set, and together with the original whole insect label as a multi-stream input to guide the network to focus on local features with significant discriminative power and suppress background interference. The results show that this biomimetic vision model outperforms the original YOLOv8n network, with improvements of 5, 1.1, and 4.6 percentage points in Recall, Precision, and mAP, respectively. Compared to embedded attention mechanisms like CBAM and SE, the biomimetic vision model’s mAP is higher by 3.3 and 2.7 percentage points, respectively. The biomimetic vision strategy in this study achieves a good balance between detection accuracy and inference cost through local saliency guidance and dual-stream feature fusion, expanding the data annotation paradigm for biomimetic small-object detection.

Sihan Huang et al. proposed a single-stage detection network BRSTD driven by bionic vision to solve the problem that it is difficult to detect remote sensing small targets under complex background and cross-scale differences [14]. The network designed a parallel pathway convolution XYWConv to simulate the antagonistic receptive field of X/Y/W cells to enhance fine-grained and contextual information. The bottom-up spatial channel attention XYWA is introduced to improve the target background discrimination, and a top-down feedback suppression attention TDSA is added between the neck and the detection head to suppress the shallow large target and background response and highlight the small target representation. The results show that this network systematically improves the model’s performance in small-object detection across multiple datasets, while maintaining extremely low parameters and computational cost, demonstrating an effective combination of biomimetic mechanisms and efficient detection.

Muhammad Akbar Andi Arief and colleagues developed a system based on chlorophyll fluorescence imaging technology to address the issue of changes in the photosynthetic efficiency of strawberries under drought conditions [15]. The system excites chlorophyll fluorescence using blue LED light and captures the fluorescence signal with a monochrome camera to measure the maximum photochemical quantum efficiency (Fv/Fm). The study shows that drought stress significantly reduces the photosynthetic efficiency of strawberries. This research not only validates the potential application of chlorophyll fluorescence imaging technology in plant drought monitoring but also provides a new technical approach for non-destructive real-time monitoring of plant health.

These studies have proposed novel agricultural intelligent monitoring solutions by incorporating biomimetic vision or deep learning technologies, demonstrating the potential application value of bio-inspired design and deep learning models in crop health monitoring, environmental sensing, and pest and disease control. However, when performing drought stress detection during the seedling stage of the Yunnan large-leaf tea variety, existing models are still prone to the influence of drought level confusion, resulting in low classification accuracy.

To accurately control the extent of drought stress in response to the above issues, this study uses a PEG-6000 solution to simulate drought conditions and constructs a dataset using chlorophyll fluorescence imaging technology [16]. Tests show that, although there are more complex factors in the field drought environment, the basic physiological effects of drought stress on tea seedlings are similar. Therefore, the phenotypic changes observed in the pot experiments are highly consistent with the results from the field experiments. With the compound eye’s parallel sampling mechanism as the core, Compound-Eye Apposition Concatenation optimization is applied during both training and inference phases to tile and concatenate the input images [17]. After inference, the concatenated image is automatically divided into sub-images based on a grid, and the predicted bounding boxes are reverse-mapped to the coordinate system of each sub-image. Based on the YOLOv13 network, the environmental information acquisition and integration mechanism of primates’ “multi-scale parallelism—global modulation—long-range integration” is simulated, and multi-scale linear attention is used to optimize the network [18,19]. Simulating the retinal wide-field lateral inhibition and cortical selective convergence mechanisms, CMUNeXt is used to optimize the network’s backbone [20,21]. To further enhance the localization accuracy of drought stress detection and accelerate model convergence, a dynamic attention process simulating peripheral search, saccadic focus, and central fovea refinement in primates is used [22]. Inner-IoU is applied for targeted improvement of the loss function [23]. This study addresses the key challenges in drought stress detection during the seedling stage of Yunnan large-leaf tea trees. The proposed improved YOLOv13-based network not only enables fast and accurate detection of drought stress responses in tea trees, laying a solid foundation for the intelligent advancement of the tea production field, but also contributes to the application of bio-inspired computing in complex ecosystems [24].

## 2. Materials and Methods

### 2.1. Data Collection

In this study, two-year-old Yunnan large-leaf tea seedlings were used as experimental materials. To reduce the influence of individual differences on the experimental results, the plant height of the selected tea seedlings was between 15 and 23 cm, the upper and lower stem diameters were basically the same, and the overall growth and individual size were relatively neat. to ensure the consistency and controllability of the growth environment, a uniform pot container with a diameter of 16 cm and a height of 16.9 cm was used. The cultivated soil was taken from the tea garden of Yunnan Agricultural University, with 0.5 kg soil per pot. The soil physical and chemical properties were determined by PR-3002-TRREC-N01 soil sensor (Shandong Sain Electronics Technology Co., Ltd., Zibo, China) [25]. The average soil pH was 4.9, and the contents of nitrogen, phosphorus and potassium were 79 mg/kg, 230 mg/kg and 223 mg/kg, respectively.

To accurately simulate the drought stress environment of tea seedling stage, this study selected 0%, 10%, 20% and 30% four concentration gradients of PEG-6000 solution to achieve different intensities of drought stress by regulating osmotic pressure. As a high molecular osmotic regulator, PEG-6000 has strong water absorption capacity, which can significantly increase the osmotic pressure of external solution and make it exceed the osmotic potential of plant cell fluid, thus inhibiting root water absorption and inducing plants to produce physiological water loss similar to natural drought conditions. To further realize the quantification and objective classification of drought degree, according to the relevant standards of ‘agricultural drought grade’ (GB/T 32136-2015) and ‘tea garden drought grade’ (DB5308/T 67-2022) [26,27], soil relative humidity was used as the classification index of drought degree, and the specific classification is shown in Table 1. To ensure that the plants adapt to the drought environment and exhibit stable physiological responses, this study maintained the normal growth of the tea seedlings for 15 days after the drought treatment and used the PR-3002-TRREC-N01 soil sensor to measure the soil moisture content of the potted plants daily at 9 AM. By precisely controlling soil moisture variation, we ensured that the soil moisture at each drought level remained within the preset range.

The phenotypic acquisition device was Plant Explorer Pro (Pheno Vation, Wageningen, The Netherlands), and the width and height of the imaging area were 53 cm and 40 cm, respectively. The image acquisition module is equipped with a 1.3 million pixel CCD camera, combined with a filter wheel and an LED excitation light source, and multiple types of optical signals can be obtained in a single measurement. The device outputs a total of 14 types of image data, as shown in Table 2, with the correlation to drought stress analyzed using Pearson correlation. The results indicate that the correlation between Maximum Photosynthetic Efficiency and drought stress is significantly higher than that of other indicators. Therefore, this study uses the $F_{v}/F_{m}$ channel images as the input data for the detection model.

### 2.2. Dataset Construction

A total of 324 original samples were collected in this study, with each sample derived from a different experimental plant. For the collected raw images, 20% were randomly selected as the validation set to assess the generalization ability of the detection model. The remaining 259 images were randomly split into training and testing sets in an 8:2 ratio.

**Figure 1:**
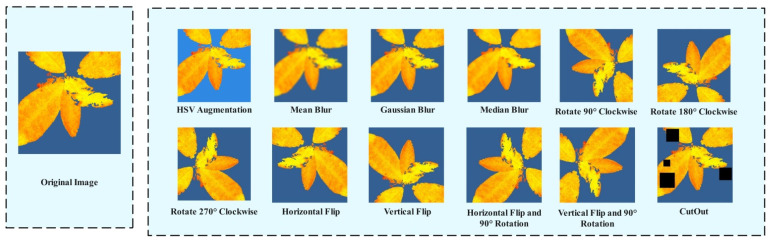
Data Augmentation.

To further enhance the model’s robustness and generalization ability, and to better extract drought stress features in tea leaves, this study applied data augmentation techniques to the entire dataset, including HSV spatial disturbance, Mean Blur, Gaussian Blur, Median Blur, CutOut, D4 (dihedral group) transformation, and random brightness transformations [28,29], as shown in Figure 1. The HSV spatial perturbation is mainly used to simulate different color temperatures, white balance, and backlighting conditions, reducing the model’s reliance on non-physiological color variations and false color mapping, while enhancing the model’s focus on drought-related features, thereby improving the model’s adaptability to different shooting conditions. Mean Blur is used to simulate the detail degradation caused by slight defocus and resampling. It preserves the large-scale intensity gradient while suppressing high-frequency noise and random particles, thereby improving the recognition accuracy of the model for low-definition images. By approximating the point spread function of the optical system, Gaussian Blur simulates the blur effect caused by defocus or slight motion, and enhances the detection accuracy of the model under the conditions of fast imaging or unstable exposure. Median Blur uses a small-scale convolution kernel for median filtering, which effectively removes salt and pepper noise and hot pixel outlier interference. While maintaining the integrity of the edge structure, it reduces the misjudgment probability of the model for abnormal bright and dark spots. CutOut is used to simulate common occlusions such as mutual occlusion of leaves, identification stickers, and shadow reflection. The number of random occlusions is set to 1–6, and the occlusion area accounts for 5–40% of the original image. On the premise of not changing the topological structure of the image, the D4 transform (rotation and mirror combination) applies rotation and mirror operation to the sample to enhance the generalization ability of the model to different shooting angles and swing habits.

**Figure 2:**
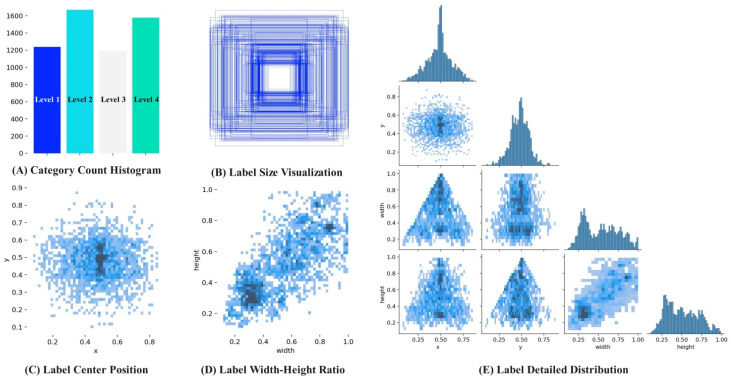
Dataset Label Visualization.

To ensure the objective independence of the test and validation sets, this study adopts the mode of ‘first division and then enhancement’ in the construction of the data set. A total of 4212 augmented images were obtained, with 2691 images in the training set, 676 images in the test set, and 845 images in the validation set. The dataset label visualization is shown in Figure 2. Figure 2A shows the histogram of the distribution of four drought stress labels. Figure 2B shows the width-height distribution of the bounding boxes, with the center of all bounding boxes set at the image center. Figure 2C illustrates the spatial distribution of the center points in the image coordinate system, while Figure 2D shows the distribution of the bounding box aspect ratios. Figure 2E provides a detailed distribution of the drought stress labels in the image [30].

### 2.3. Compound Eye Apposition Concatenation Optimization

As a multi-aperture visual organ found in insects and crustaceans, the compound eye consists of densely arranged small ommatidia in a hexagonal honeycomb pattern. To expand the field of view, bees, while hovering to collect nectar, synchronize the sampling of each ommatidium along slightly angled optical axes. The forward acute region prioritizes analyzing details of the flower crown and color contrast, while the lateral ommatidia parallelly sense background optical flow to help stabilize posture. Similarly, dragonflies perform similar operations when hunting in the air. The large dorsal region samples the upper front field in parallel, aiding in target locking. The lateral and ventral ommatidia synchronize to monitor wide-area motion cues, forming a division of labor for wide-angle coverage and local refinement. This is also the parallel sampling mechanism of the compound eye, which does not perform cross-view fusion early on. Instead, it provides inputs for subsequent neural integration and behavioral decision-making by maintaining high directional resolution and coverage range [17].

To maximize the inference speed of the model, this study centers on the compound eye’s parallel sampling mechanism, incorporating Compound-Eye Apposition Concatenation optimization during both training and inference phases to tile and concatenate the input images. Since YOLO series algorithms automatically process the input image to a size of 640 × 640 pixels, the number of sub-images in the tiled concatenated image is x^2^, where x represents the number of sub-images in the horizontal direction. After inference, Compound-Eye Apposition Concatenation automatically divides the concatenated image into sub-images based on a grid and reverse-maps the predicted bounding boxes to the coordinate system of each sub-image. During the splitting process, this study first calculates the absolute values of the normalized TXT labels, as shown in Equations (1)–(4). Here, $a_{orig}$ and $b_{orig}$ represent the coordinates of the center of the bounding box, $w_{orig}$ and $h_{orig}$ represent the width and height of the bounding box, and $W_{orig}$ and $H_{orig}$ represent the width and height of the combined image. After the image is split, the absolute labels are normalized and restored according to the size of the cropped image, as shown in Equations (5)–(8). Here, $c_{start}^{\left(i,j\right)}$ and $d_{start}^{\left(i,j\right)}$ represent the coordinates of the top-left corner of the current cropped image, while $W_{tile}$ and $H_{tile}$ represent the width and height of the cropped image.

(1) $$a_{abs}=a_{orig}\times W_{orig}$$

(2) $$b_{abs}=b_{orig}\times H_{orig}$$

(3) $$w_{abs}=w_{orig}\times W_{orig}$$

(4) $$h_{abs}=h_{orig}\times H_{orig}$$

(5) $$a=\frac{a_{abs}-c_{start}^{\left(i,j\right)}}{W_{tile}}$$

(6) $$b=\frac{b_{abs}-d_{start}^{\left(i,j\right)}}{H_{tile}}$$

(7) $$w=\frac{w_{abs}}{W_{tile}}$$

(8) $$h=\frac{h_{abs}}{H_{tile}}$$

As shown in Table 3, the Compound-Eye Apposition Concatenation optimization test results indicate that when the number of concatenated sub-images is 9 (x = 3), the average detection time of the model is reduced to 12.20% of the single-image input, with only a 2% decrease in mAP@50. Compared to current mainstream inference acceleration schemes such as Dynamic Convolution, GhostNetV2, and Sparse Pyramid Transformer, while CEAC may slightly reduce the detection accuracy of the original network, the overall mAP@50 drop remains within 2% when the number of concatenated sub-images is 16 (x ≤ 3), with no impact on the integration of other inference speed optimization strategies. When the number of sub-images exceeds 16 (x ≥ 4), the model’s accuracy begins to significantly decline, with an overall drop exceeding 4%. Considering the trade-off between speed and accuracy, this study defaults to using 9 (x = 3) as the number of concatenated sub-images.

### 2.4. YOLOv13 Network Improvement

As one of the latest representatives in the field of object detection, the YOLOv13 network continues the core design philosophy of the YOLO series for end-to-end real-time detection [31]. It also introduces the Full-Pipeline Aggregation-and-Distribution paradigm based on Hypergraph-based Adaptive Correlation Enhancement. By distributing the correlation-enhanced features across the entire network, it effectively achieves fine-grained information flow and representation synergy throughout the network. However, in the drought stress detection task, drought phenotypes often appear as fragmented weak fluorescence spots, narrow weak fluorescence bands along the leaf veins, or local fluorescence decrease at the leaf edges. The discriminative cues that determine the stress level are often diluted in low-contrast, small-scale textures, making it prone to missed detections and level confusion. Additionally, the YOLOv13 network tends to segment patchy and striped patterns into local fragments, making it difficult to effectively capture long-range associations across the leaf and veins, which results in confusion between adjacent levels at decision boundaries.

**Figure 3:**
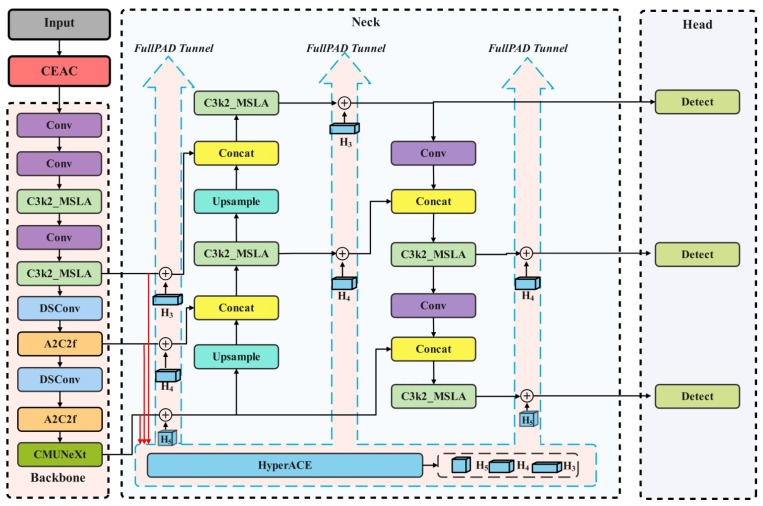
MC-YOLOv13-L Network Structure.

To address the above issues, this study simulates the environmental information acquisition and integration mechanism of primates, “multi-scale parallelism—global modulation—long-range integration,” and introduces a multi-scale linear attention mechanism to optimize the Backbone and Neck parts of the YOLOv13 network. The method uses cross-scale parallel extraction of details and overall morphology, with global aggregation and position-dependent redistribution to explicitly model long-range dependencies. Drawing from the retinal wide-field lateral inhibition and cortical selective convergence mechanisms, the CMUNeXt Block is added to the Backbone layer. This enhances the model’s feature discriminative ability without increasing resolution-related overhead and improves the information carrying capacity of the single channel. To further improve the localization accuracy of drought stress detection and accelerate model convergence, a dynamic attention process simulating peripheral search, saccadic focus, and central fovea refinement in primates is used. Inner-IoU is applied for targeted improvements to the YOLOv13 network’s loss function. The improved MC-YOLOv13-L network structure is shown in Figure 3, with detailed parameters listed in Table 4. Here, M and C represent the multi-scale linear attention mechanism and CMUNeXt Block, respectively, while L refers to the loss function optimization.

#### 2.4.1. Multi-Scale Linear Attention Mechanism Optimization

In drought stress detection of Yunnan large-leaf tea seedlings, the imaging scale of the leaves in the compound eye-stitched images is often small. Drought-related phenotypes often appear as fragmented weak fluorescence spots, narrow weak fluorescence bands along the leaf veins, or localized fluorescence intensity decrease at the leaf edges. In such cases, the discriminative cues that determine the stress level are often diluted in low-contrast, small-scale textures, making it easy to miss detections and confuse levels. Additionally, due to individual plant variation, there is overlap between phenotypes of different drought stress levels, and it is difficult to stably differentiate levels based solely on local neighborhood evidence.

**Figure 4:**
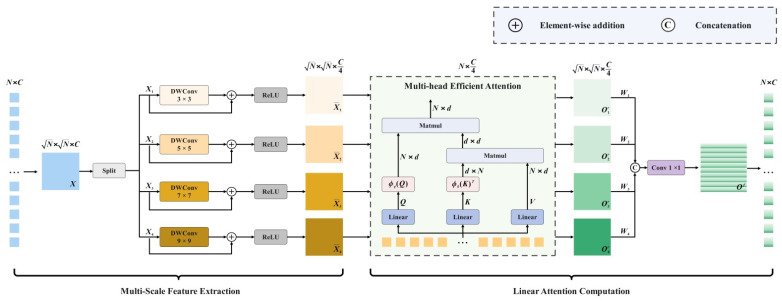
Multi-Scale Linear Attention Mechanism.

In the natural world, primates’ environmental information acquisition and integration follow a “multi-scale parallelism—global modulation—long-range integration” pattern. From the retina to the primary visual cortex, pathways with different receptive field scales parallelly encode high and low spatial frequency information. This is followed by lateral and surround inhibition, which enhances local contrast on one hand, while constraining overall gain on the other. Long-range horizontal connections between the primary and secondary visual cortices tend to integrate directionally coherent discrete segments into continuous structures on a larger spatial scale [18]. Drawing from the above biological model, this study adopts a multi-scale linear attention mechanism to specifically optimize the YOLOv13 network, as shown in Figure 4. This optimization employs parallel multi-scale paths that simultaneously capture heterogeneous information, ranging from fragmented weak fluorescence spots to whole leaf morphology and vein orientation [19]. Then, through global aggregation and position-dependent redistribution, it aligns and collaborates cross-region and multi-scale evidence at the same level. This significantly enhances the visibility and consistency of weak signals along the leaf veins and local attenuation at the leaf edges. Finally, learnable channel re-calibration is introduced to achieve information compression and discriminative subspace projection without changing the spatial resolution, reducing missed detections and alleviating drought level confusion.

The processing of input features is shown in Equation (9). The Multi-Scale Linear Attention first restores them into a 2D feature map and divides them into four blocks along the channels. Each block is then subjected to depthwise separable convolutions with kernel sizes of 3 × 3, 5 × 5, 7 × 7, and 9 × 9. After performing element-wise residual addition on each branch, ReLU is used for non-linear activation. The core idea is to preserve fine boundary information with small-scale convolutions and represent overall morphology with large-scale convolutions.

(9) $${\bar{X}}_{i}=ReLU(f_{k_{i}\times k_{i}}^{dwc}\left(X_{i}\right)+X_{i})$$

For the output after non-linear activation, ${\bar{X}}_{i}\in R^{\sqrt{N}\times \sqrt{N}\times \frac{C}{4}}$, MSLA first reshapes it into ${\bar{X}}_{i}^{r}\in R^{N\times \frac{C}{4}}$ and applies Multi-Scale Efficient Attention to extract global features. As shown in Equations (10)–(12), where $Q_{i,h},\;K_{i,h}$, and $V_{i,h}$ represent the query, key, and value of the i-th branch and h-th head, respectively. $W_{i,h}^{q},\;W_{i,h}^{k}$, and $W_{i,h}^{v}$ represent the projection weight matrices for the query, key, and value, respectively. In Multi-Scale Efficient Attention, $\phi_{q}(Q)$ applies the softmax function to each row of Q, primarily used to identify the feature channels that the model focuses on more. $\phi_{k}(K)$ applies the SoftMax function to each column of K, used to find the positions that better express the drought stress features. After aggregating K and V to obtain a global representation, interaction with Q allows for the effective avoidance of constructing an N × N attention matrix, thereby reducing computational complexity.

(10) $$Q_{i,h}={\bar{X}}_{i}^{r}W_{i,h}^{q}$$

(11) $$K_{i,h}={\bar{X}}_{i}^{r}W_{i,h}^{k}$$

(12) $$V_{i,h}={\bar{X}}_{i}^{r}W_{i,h}^{v}$$

After each scale branch completes linear attention and multi-head concatenation, followed by the output of linear mapping, a sequence output $O_{i}\in R^{N\times \frac{C}{4}}$ is obtained. To enhance the network’s multi-scale feature fusion capability, this output will be reshaped into $O_{i}^{r}\in R^{\sqrt{N}\times \sqrt{N}\times \frac{C}{4}}$. The model’s fusion process is shown in Equation (13), where $f_{1\times 1}$ represents a 1 × 1 convolution, and $w_{i}$ denotes the learnable parameters.

(13) $$O^{f}=f_{1\times 1}([w_{1}O_{1}^{r},w_{2}O_{2}^{r},w_{3}O_{3}^{r},w_{4}O_{4}^{r}])$$

#### 2.4.2. CMUNeXt Block Optimization

In the task of drought stress intensity grading during the seedling stage of tea trees, the YOLOv13 network tends to segment the patchy and striped distributions caused by drought into local fragments, making it difficult to effectively capture long-range associations across the leaf and veins, resulting in confusion between adjacent levels at decision boundaries. Additionally, YOLOv13 adjusts the input images to a resolution of 640 × 640 pixels, which reduces the resolution of the drought features after compound eye stitching. When processing low-resolution feature maps, the YOLOv13 network’s reconstruction of fine-grained stress boundaries is still limited by local inductive bias, making it prone to missed detections and unstable drought stress level boundaries.

**Figure 5:**
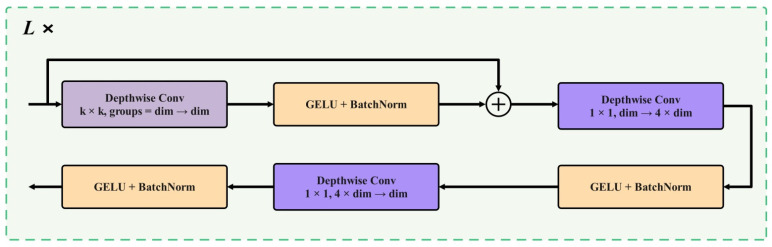
CMUNeXt Module.

In the primate visual system, after incident light is converted into neural activity in the retina, horizontal cells and amacrine cells implement lateral inhibition and integration across a wide field, suppressing random noise and enhancing robust structural features such as edges and patches. Meanwhile, the inhibitory processed signals are incrementally added to the original input. Once reaching the primary visual cortex, neurons form high-dimensional and sparse feature representations across dimensions such as orientation and spatial frequency. Later, in higher cortical areas, neurons perform selective aggregation and simplified readout of high-dimensional descriptions, thereby generating compact representations relevant to the task [21]. Inspired by this visual model, this study adds the CMUNeXt Block at the end of the backbone network for optimization [20], as shown in Figure 5.

CMUNeXt Block first performs spatial filtering in each channel with large kernel deep convolution, and integrates long-distance spatial relationships with lower parameters and computational overhead. GELU and BatchNorm are used to stabilize the numerical range and suppress noise. By adding residuals to the input, CMUNeXt Block can retain the original path while introducing wide-area information, alleviate gradient attenuation and avoid excessive disturbance to the representation. Two subsequent 1 × 1 Depthwise Convs were used for channel recombination. The first Depthwise Conv extends the number of channels from the original scale to a higher dimension, so that the spatial evidence previously obtained by large kernel deep convolution can be flexibly combined and screened in a wider channel space. The second Depthwise Conv pushes the channel count back to the original scale and feeds back to the downstream level in a compact form. After the two Depthwise Conv, GELU and BatchNorm are equipped to enhance the nonlinear expression while maintaining the stability of the distribution. This design can improve the feature discrimination of the model without increasing the resolution-related overhead, and improve the information carrying capacity of the single channel.

The specific calculation process is shown in Equations (14)–(16). Among them, $f_{l-1}$ represents the input characteristics of the module, $\sigma_{1}$ for the GELU activation function, and BN represents batch normalization (BatchNorm) operation. $f_{l}^{\prime }$ is the output after the residual connection, $f_{l}^{\prime \prime }$ is the output after expanding the channel number from the original dimension to the high-dimensional output, and $f_{l}$ is the final output of the CMUNeXt Block.

(14) $$f_{l}^{\prime }=BN\left(\sigma_{1}\left\{DepthwiseConv\left(f_{l-1}\right)\right\}\right)+f_{l-1}$$

(15) $$f_{l}^{\prime \prime }=BN\left(\sigma_{1}\left\{PointwiseConv\left(f_{l}^{\prime }\right)\right\}\right)$$

(16) $$f_{l}=BN\left(\sigma_{1}\left\{PointwiseConv\left(f_{l}^{\prime \prime }\right)\right\}\right)$$

#### 2.4.3. Auxiliary Bounding Box Algorithm Optimization

As a high-performance target detection algorithm, YOLOv13 has excellent performance in real-time and detection accuracy. However, when it is directly applied to the drought stress detection task of tea seedlings, there are limitations such as slow convergence speed of the model and limited ability to perceive local features of leaf occlusion areas. In contrast, when monkeys search for mature fruits on trees with dense branches and leaves, their visual system typically relies on peripheral retinal cues for wide-area search retrieval. Then, the fixation point is accurately moved to the vicinity of the target with a small saccade, and the attention window is narrowed to a smaller area. At this point, the high-density cone cells in the fovea provide high resolution and color details, fruit stalk and fruit surface texture can be more clearly analyzed and distinguished. In this way, through dynamically adjusting the attention region and receptive field scale, the visual system enables robust differentiation and precise localization of adjacent fruits and backgrounds in complex and overlapping natural environments [22].

**Figure 6:**
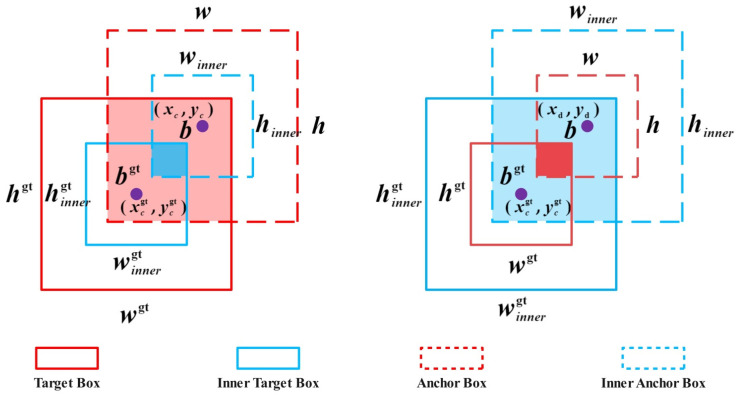
Auxiliary Bounding Box Algorithm Optimization.

To further improve the localization accuracy of drought stress detection in tea seedlings and accelerate model convergence, this study, inspired by primate visual strategies, introduces Inner-IoU to modify the loss function of the YOLOv13 network [23], as shown in Figure 6. A scale factor ratio is introduced to adjust the size of the auxiliary bounding boxes, thereby altering their impact on the regression process. For low IoU scenarios during the early stages of training and in cases of strong occlusion, larger auxiliary boxes are used to geometrically expand the potential intersection area, increasing the effective regression range. As the model trains, the predicted bounding boxes gradually approach the true bounding boxes, and the size of the auxiliary bounding boxes is dynamically reduced. This improves the IoU’s sensitivity to fine-scale differences, thereby enhancing localization accuracy and accelerating model convergence.

In Figure 6, the Target Box refers to the actual bounding box of an object in the image, the Anchor Box refers to the prior box in the image, and the Inner Target Box and Inner Anchor Box are the new bounding boxes obtained by adjusting the true target box and anchor box according to a scaling factor. The centers of the Target Box and Inner Target Box are $b^{gt}$ and $\left(x_{c}^{gt},y_{c}^{gt}\right)$, respectively, while the centers of the Anchor Box and Inner Anchor Box are b and $\left(x_{c},y_{c}\right)$, with width and height represented by w and h, respectively. The calculation of the Inner Target Box is shown in Equations (9) and (10), where $b_{l}^{gt}$ and $b_{r}^{gt}$ represent the left and right boundary coordinates, and $b_{t}^{gt}$ and $b_{b}^{gt}$ represent the top and bottom boundary coordinates. The calculation of the Inner Anchor Box is shown in Equations (17)–(20), where $b_{l}$ and $b_{r}$ represent the left and right boundary coordinates of the Inner Anchor Box, and $b_{t}$ and $b_{b}$ represent the top and bottom boundary coordinates.

(17) $$b_{l}^{gt}=x_{c}^{gt}-\frac{w^{gt}\times ratio}{2},b_{r}^{gt}=x_{c}^{gt}+\frac{w^{gt}\times ratio}{2}$$

(18) $$b_{t}^{gt}=y_{c}^{gt}-\frac{h^{gt}\times ratio}{2},b_{b}^{gt}=y_{c}^{gt}+\frac{h^{gt}\times ratio}{2}$$

(19) $$b_{l}=x_{c}-\frac{w\times ratio}{2},b_{r}=x_{c}+\frac{w\times ratio}{2}$$

(20) $$b_{t}=y_{c}-\frac{h\times ratio}{2},b_{b}=y_{c}+\frac{h\times ratio}{2}$$

The intersection area of the predicted box and the ground truth box is calculated as shown in Equation (21). It is mainly derived by calculating the maximum and minimum values of the left, right, top, and bottom boundaries of the two boxes to determine the width and height of the intersection area. The union is calculated as shown in Equation (14), which refers to the total area of the Inner Target Box and Inner Anchor Box minus the area of their intersection. The improved loss function is calculated as shown in Equations (22)–(25), where IoU represents the overlap between the predicted box and the ground truth box, B represents the predicted box, and $B^{gt}$ represents the ground truth box. ρ represents the Euclidean distance function, c represents the diagonal length of the minimum bounding rectangle that encloses the predicted and ground truth boxes, and α is the weighting factor used to adjust the influence of v. v represents the aspect ratio consistency measure, which is used to assess the difference in aspect ratio between the predicted box and the ground truth box.

(21) $$inter=(\min\left(b_{r}^{gt},b_{r}\right)-\max\left(b_{l}^{gt},b_{l}\right))\times (\min\left(b_{b}^{gt},b_{b}\right)-\max\left(b_{t}^{gt},b_{t}\right))$$

(22) $$union=\left(w^{gt}\times h^{gt}\right)\times {\left(ratio\right)}^{2}+\left(w\times h\right)\times {\left(ratio\right)}^{2}-inter$$

(23) $$L_{Inner-CIoU}=L_{CIoU}+IoU-\frac{inter}{union}$$

(24) $$IoU=\frac{\left\lceil B\cap B^{gt}\right\rceil }{\left|B\cup B^{gt}\right|}$$

(25) $$L_{CIoU}=1-IoU+\frac{\rho^{2}\left(b,b^{gt}\right)}{c^{2}}+\alpha v$$

### 2.5. Model Evaluation Metrics

To evaluate the performance of the MC-YOLOv13-L network in drought stress detection of tea seedlings, this study uses Precision, Recall, F1, AP@50, mAP@50, and mAP@0.5-95 to assess the model’s performance, as shown in Equations (26)–(30) [32]. Precision represents the proportion of targets predicted to belong to a specific drought stress level that actually belong to that level. Recall represents the proportion of true drought stress targets that the model successfully detects. Recall represents the proportion of true drought stress targets that the model successfully detects. Here, $T_{P}$ represents the number of targets correctly detected as drought stress, $F_{P}$ represents the number of targets incorrectly identified as drought stress, and $F_{N}$ represents the number of drought stress targets not detected by the model. AP@0.5 represents the area under the PR curve (with Recall on the x-axis and Precision on the y-axis) when the IoU threshold is set to 0.5. mAP@50 is the average of AP@0.5 for different drought levels, used to provide a comprehensive reflection of the model’s overall performance in the drought stress detection task for tea seedlings. Similarly, mAP@0.5-95 represents the AP across all thresholds from an IoU of 0.5 to 0.95, with a threshold step of 0.05.

(26) $$Precision=\frac{T_{P}}{T_{P}+F_{P}}\;$$

(27) $$\;\;Recall=\frac{T_{P}}{T_{P}+F_{N}}\;$$

(28) $$\;F1=2\times \frac{Precision\times Recall}{Precision+Recall}\;$$

(29) $$AP=\sum_{i=1}^{n-1}\left(r_{i+1}-r_{i})P_{inter}(r_{i}+1\right)$$

(30) $$mAP=\frac{\sum_{i=1}^{k}{AP}_{i}}{k}$$

## 3. Results and Analysis

To analyze the performance of the improved MC-YOLOv13-L network in the drought stress detection task for Yunnan large-leaf tea seedlings, six comparison experiments were designed. These experiments used six object detection networks: MC-YOLOv13-L, YOLOv13, YOLOv10, SSD, RT-DETR, and Faster-RCNN, and were conducted on the same dataset for model training and testing [33,34,35]. To ensure the reproducibility and rigor of the drought stress experiment results for Yunnan large-leaf tea seedlings, the software and hardware environment of this study were kept consistent, as shown in Table 5.

To control for confounding factors and ensure the rigor of model comparisons, the parameters used in the model comparison experiments are the same, as shown in Table 6. In terms of optimizer selection, the SGD optimizer was used with a momentum of 0.937 and weight decay of 0.001 [36].

### 3.1. Model Result Analysis

**Figure 7:**
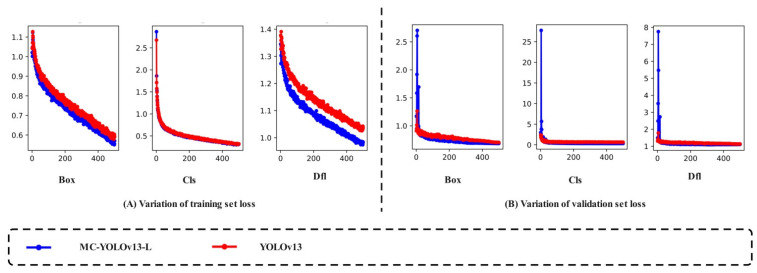
Loss Value Changes.

As one of the core components of object detection algorithm performance, the loss function is primarily used to measure the deviation between the model’s predicted results and the true annotations. In the drought stress detection task for Yunnan large-leaf tea seedlings, the smaller the loss value, the higher the match between the predicted bounding box and the true annotation of the drought-affected tea seedlings, and the better the model’s detection performance [37]. As shown in Figure 7, in the training set, MC-YOLOv13-L’s Box Loss, Cls Loss, and DFL Loss stabilized below 0.56, 0.31, and 0.98, respectively. Compared to the YOLOv13 network’s 0.59, 0.32, and 1.03, these values decreased by 5.08%, 3.13%, and 4.85%, respectively. In the validation set, MC-YOLOv13-L’s Box Loss, Cls Loss, and DFL Loss stabilized below 0.69, 0.38, and 1.10, respectively. Compared to the YOLOv13 network’s 0.71, 0.41, and 1.14, these values decreased by 2.82%, 7.32%, and 3.51%, respectively. The research results show that the MC-YOLOv13-L network’s loss function converges faster and is more stable overall. It has higher expressive ability in recognizing boundary features and texture details of drought-affected tea seedlings, effectively reducing misclassification between samples of different stress levels.

**Figure 8:**
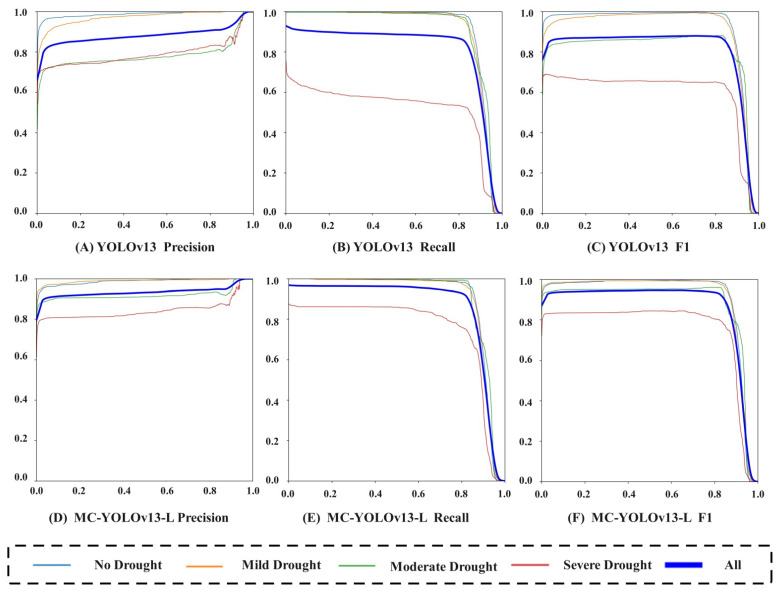
Precision, Recall, F1 Comparison.

As shown in Figure 8, MC-YOLOv13-L reached 93.03%, 95.01% and 94.01% in terms of accuracy, recall rate and balance score (F1 score), respectively. Compared with the original YOLOv13 model (88.39%, 88.08%, 88.23%), it increased by 4.64, 6.93 and 5.78 percentage points, respectively. In the original YOLOv13 network, the recall for severe drought is significantly lower than for the other three drought levels. The test results indicate that this phenomenon is mainly due to the significant wilting of tea seedling leaves under severe drought conditions, which leads to a noticeable reduction in the visible phenotypic area in chlorophyll fluorescence imaging. After processing with Compound-Eye Apposition Concatenation, the phenotypic area further shrinks, resulting in a lower recall. Compared to the original network, the improved MC-YOLOv13-L network shows a significant increase in recall for severe drought phenotype detection.

**Figure 9:**
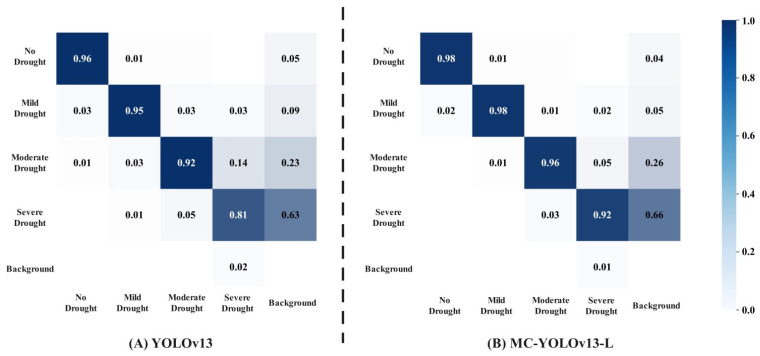
Confusion matrix comparison chart.

In the study of drought stress detection for Yunnan large-leaf tea seedlings, the confusion matrix is used to show the model’s detection performance across different drought levels. As shown in Figure 9, the rows of the matrix represent the true drought levels, while the columns represent the predicted drought levels. The larger the values on the diagonal, the higher the recognition accuracy for that class. The off-diagonal elements reflect the degree of confusion between different drought levels in the model. The results show that moderate drought and severe drought are the most easily confused. Testing revealed that this is due to similar phenotypic changes in the leaves of some tea seedlings under moderate and severe drought conditions, lacking clear boundaries. Compared to the YOLOv13 network, the improved MC-YOLOv13-L increased detection accuracy for tea seedling phenotypes under no drought stress by 2 percentage points, for mild drought stress by 3 percentage points, for moderate drought stress by 4 percentage points, and for severe drought stress by 11 percentage points. MC-YOLOv13-L effectively enhances the model’s feature discriminative ability, alleviating confusion between drought levels.

### 3.2. Ablation Study

To verify the effectiveness of the MC-YOLOv13-L network in the drought stress detection task for Yunnan large-leaf tea seedlings, and to evaluate the gains from the multi-scale linear attention mechanism, CMUNeXt module, and auxiliary bounding box algorithm optimization on the YOLOv13 network, an ablation study was conducted based on the constructed dataset. Each configuration was trained five times with different random seeds. Since in practical work, the model with the best detection results is usually selected as the final model, all parameters except for Avg-mAP are the test results of the group with the highest mAP value. As shown in Table 7, the multi-scale linear attention mechanism optimization improved the original network’s Precision by 0.55%, Recall by 3.76%, mAP@50 by 0.75%, mAP@50-95 by 1.29%, and FPS by 2.27, while reducing the original network’s FLOPs by 0.2G. This optimization reduced the original network’s missed detections and drought level confusion to some extent, while improving the model’s detection speed. The CMUNeXt module optimization improved the original network’s Precision by 0.63%, Recall by 4.13%, mAP@50 by 1.36%, mAP@50-95 by 1.41%, and FLOPs by 0.9G, while reducing the original network’s FPS by 1.23. Although this optimization reduced the model’s detection speed by 2.20%, it significantly improved the model’s feature discriminative ability. The auxiliary bounding box algorithm optimization, without changing the original network’s basic architecture, improved the original network’s Precision by 0.72%, Recall by 3.47%, mAP@50 by 1.08%, and mAP@50-95 by 1.36%, significantly improving the localization accuracy of tea seedling drought stress detection and recall rate. After the overall improvements, compared to the original YOLOv13 network, the MC-YOLOv13-L network’s Precision, Recall, mAP@50, and mAP@50-95 were improved by 4.64%, 6.93%, 4.2%, and 5.07%, respectively, with only a 0.63 decrease in FPS.

**Figure 10:**
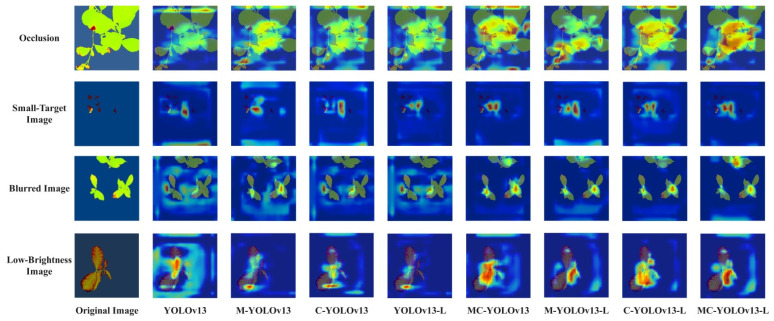
Grad-CAM Heatmap.

To verify the performance improvement of the multi-scale linear attention mechanism, CMUNeXt module, and auxiliary bounding box algorithm optimization on the YOLOv13 network, a visual analysis of the model’s attention regions was conducted. This study further introduces Grad-CAM (Gradient-weighted Class Activation Mapping) for analysis [38]. As a gradient-based visualization method, Grad-CAM is primarily used to explain the decision-making process of deep convolutional neural networks in object detection tasks. It calculates the gradient information from specific convolutional layers in the network and generates class activation heatmaps to visually display the key regions the model focuses on when predicting specific categories. In this study, the highlighted areas focused on by the model are all phenotypic features related to drought stress, such as wilting at the leaf edges and changes in color. In this study, the highlighted areas focused on by the model are all phenotypic features related to drought stress, such as wilting at the leaf edges and changes in color. The analysis results are shown in Figure 10, indicate that the improved YOLOv13 network shows enhanced attention to details under conditions such as occlusion, small objects, blur, and low illumination, with significantly improved region focusing capability.

### 3.3. Model Comparison Experiments

In the drought stress detection task for Yunnan large-leaf tea seedlings, the performance of the model directly determines the accuracy and stability of practical application. To comprehensively evaluate the detection performance of the improved MC-YOLOv13-L in practical tasks, this study selected MC-YOLOv13-L, YOLOv13, YOLOv10, SSD, RT-DETR, Faster-RCNN six target detection networks to carry out comparative experiments. The results are shown in Table 8. Compared to the original YOLOv13 network, YOLOv10, SSD, RT-DETR, and Faster-RCNN, the MC-YOLOv13-L network improved Precision by 4.64%, 6.4%, 17.66%, 6.68%, and 17.79%, respectively. Recall was improved by 6.93%, 7.72%, 14.73%, 10.08%, and 18.99%, respectively. F1 increased by 5.78%, 7.05%, 16.26%, 8.38%, and 18.38%, respectively. mAP@50 was improved by 4.2%, 5.29%, 14.59%, 7.19%, and 17.32%, respectively.

**Figure 11:**
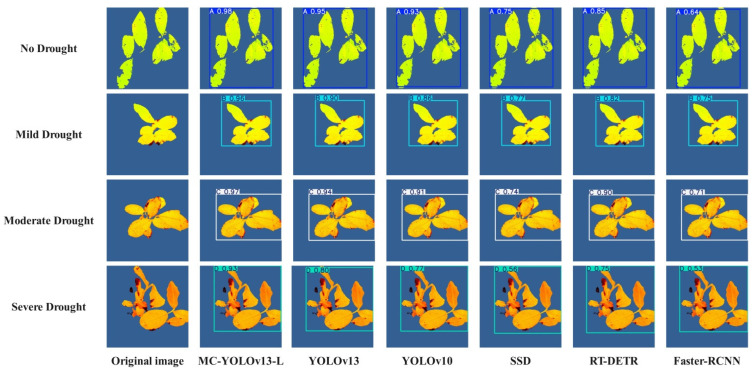
External Validation.

To evaluate the robustness and adaptability of the MC-YOLOv13-L network in the drought stress detection task of Yunnan large-leaf tea seedlings, this study further compared and analyzed the detection performance of different networks under four drought conditions: No Drought, Mild Drought, Moderate Drought, and Severe Drought. The typical comparison results are shown in Figure 11. In order to ensure the objectivity of the evaluation process, this study used independent samples for external verification. All experimental tea seedling samples were collected from the Laobanzhang base in Xishuangbanna, Yunnan Province. The external validation set consists of 100 samples, with 25 samples for each drought stress level. The drought treatment and data collection equipment are the same as those used in the training set, and this dataset was not involved in the training process. The results show that, compared to YOLOv13, the MC-YOLOv13-L network’s phenotype detection performance improved under all four stress conditions, with the most significant improvement observed under Severe Drought stress. The improved MC-YOLOv13-L network provides reliable technical support for the intelligent recognition and monitoring of Yunnan large-leaf tea seedlings in drought stress environments.

## 4. Conclusions and Discussion

In the drought stress detection task for Yunnan large-leaf tea seedlings, this study used the Plant Explorer Pro as the data collection device. Compared to traditional physiological indicator detection, the chlorophyll fluorescence imaging technology used by the Plant Explorer Pro can sensitively reflect the phenotypic changes in tea seedling leaves under mild drought conditions. To maximize the model’s detection speed, this study uses the compound eye’s parallel sampling mechanism as the core, incorporating Compound-Eye Apposition Concatenation optimization during both training and inference phases to tile and concatenate the input images. After inference, the concatenated image is automatically divided into sub-images based on a grid, and the predicted bounding boxes are reverse-mapped to the coordinate system of each sub-image. Addressing the inherent limitations of the YOLOv13 network in detecting fine-grained weak fluorescence along leaf veins and long-range dependency phenotypes, this study simulates the environmental information acquisition and integration mechanism of primates’ “multi-scale parallelism—global modulation—long-range integration” and optimizes the network’s Backbone and Neck using multi-scale linear attention. This allows for information compression and discriminative subspace projection without changing the spatial resolution, reducing missed detections and alleviating drought level confusion. The YOLOv13 network’s reconstruction of fine-grained stress boundaries is limited by local inductive bias, which leads to missed detections and unstable drought stress level boundaries. Inspired by the retinal wide-field lateral inhibition and cortical selective convergence mechanisms, CMUNeXt is used to optimize the network’s Backbone, thereby enhancing the model’s feature discriminative ability without increasing resolution-related overhead and improving the information carrying capacity of a single channel. Simulating the dynamic attention process of primates’ peripheral search, saccadic focus, and central fovea refinement, the loss function of the network is optimized using Inner-IoU. This improves the localization accuracy and accelerates model convergence. The results show that:

(1) Compared with YOLOv13 network, Box Loss, Cls Loss, and DFL Loss of MC-YOLOv13-L network decreased by 5.08%, 3.13%, and 4.85%, respectively, in the training set and decreased by 2.82%, 7.32%, and 3.51%, respectively, in the validation set. Furthermore, the improved network demonstrated faster convergence, enhanced stability, and stronger generalization capability.
(2) Ablation experiments show that the multi-scale linear attention mechanism optimization results in improvements of 0.55% for Precision, 3.76% Recall, 0.75% mAP@50 and 2.27 FPS for the YOLOv13 network on the basis of reducing 0.2G FLOPs. CMUNeXt module optimization results in improvements of 0.63% for Precision, 4.13% Recall and 1.36% mAP@50 for the original network, and results in a reduction of 1.23 FPS. The auxiliary bounding box algorithm optimization results in improvements of 0.72% for Precision, 3.47% Recall and 1.08% mAP@50 for the original network without changing the basic architecture of the original network. Compared with YOLOv13, the overall improved MC-YOLOv13-L network has an improved accuracy, recall rate and mAP@50 of 4.64%, 6.93% and 4.2%, respectively, on the basis of only reducing FPS by 0.63. Although the complexity of the model increased the computational load, the FPS of the MC-YOLOv13-L model only decreased by 0.63, still maintaining a high inference speed.
(3) The model comparison experiment shows that, compared to the original YOLOv13 network, YOLOv10, SSD, RT-DETR, and Faster-RCNN, the MC-YOLOv13-L network improved Precision by 4.64%, 6.4%, 17.66%, 6.68%, and 17.79%, respectively. Recall increased by 6.93%, 7.72%, 14.73%, 10.08%, and 18.99%, respectively. F1 improved by 5.78%, 7.05%, 16.26%, 8.38%, and 18.38%, respectively. mAP@50 increased by 4.2%, 5.29%, 14.59%, 7.19%, and 17.32%, respectively. The external validation results show that the improved MC-YOLOv13-L network can quickly and accurately capture the drought resistance response of tea seedlings under mild drought conditions, and its detection accuracy is significantly better than mainstream algorithms.

The MC-YOLOv13-L network developed in this study not only provides technical support and methodological innovation for the digital management of plateau characteristic agricultural industry but also contributes to the application transformation of bio-inspired computing in complex ecosystems to some extent. The developed drought stress detection model can help farmers adjust irrigation plans more promptly, preventing tea seedling growth limitations or damage caused by drought stress.

However, it should be noted that this study focuses solely on Yunnan large-leaf tea as the research subject, and the simulation of the compound eye mechanism is limited to parallel visual stitching, without achieving dynamic regulation of bio-inspired optimization. Additionally, the advantages of MC-YOLOv13-L are more evident in the specific task of drought stress detection for Yunnan large-leaf tea seedlings, but in some more generalized detection tasks, its performance is not significantly superior to the original YOLOv13 network. In the future, our team will expand the research subjects to different regions and crop types, and conduct large-scale cross-regional validation to enhance the generalization ability and transferability of the method. At the same time, we will develop compound-eye-like multi-scale stitching techniques to adaptively regulate the size and number of sub-image blocks. PEG-induced drought treatment cannot fully simulate the complexity of natural field drought, and the canopy structure of tea trees in tea gardens is more complex than in laboratory environments, with more background interference. Therefore, our team will conduct field experiments in the future to measure plant phenotypes under varying lighting conditions and environmental backgrounds. A lightweight design will be employed to optimize the model’s inference speed and computational resource consumption to meet the requirements for real-time detection. By incorporating adaptive optimization techniques, the model’s generalization ability and adaptability to different drought levels, crops, and complex backgrounds will be improved.

## Figures and Tables

**Table 1:** Drought grade division.

Drought Severity	Soil Relative Humidity (W)	Drought Grade	Impacts and Manifestations
No Drought	W ≥ 60%	Level 1	Soil moisture is adequate, and tea plants grow normally.
Mild Drought	50% ≤ W < 60%	Level 2	The surface soil begins to dry, and the leaves show slight wilting.
Moderate Drought	40% ≤ W < 50%	Level 3	The surface and parts of the deeper soil layers are dry, and some leaves turn yellow.
Severe Drought	30% ≤ W < 40%	Level 4	Soil moisture is severely deficient, with a large number of leaves wilting and falling, and some branches drying out and dying.

**Table 2:** Types of spectral imaging.

ID	Symbol	Image Type	Correlation with Drought Stress
1	$F_{0}$	Basic Fluorescence, Minimal Fluorescence	0.160
2	$F_{m}$	Maximum Fluorescence	0.421
3	$F_{v}/F_{m}$	Maximum Photosynthetic Efficiency	0.890
4	qL	Photochemical Quenching Coefficient(Lake Model Based)	0.209
5	qP	Photochemical Quenching Coefficient(Swamp Model Based)	0.345
6	qN	Non-Photochemical Quenching Coefficient (rarely applied in field conditions)	0.295
7	NPQ	Non-Photochemical Quenching Coefficient (widely applied in field conditions)	0.001
8	$\emptyset_{PSII}$	Effective Quantum Yield of Photosystem II	0.550
9	NDVI	Normalized Difference Vegetation Index	0.338
10	$\emptyset_{NO}$	Quantum Yield of Non-Regulated Energy Dissipation	0.597
11	$\emptyset_{NPQ}$	Quantum Yield of Regulated Energy Dissipation	0.173
12	Chl−Idx	Chlorophyll Index	0.222
13	Ari−Idx	Anthocyanin Reflectance Index	0.181
14	ETR	Electron Transport Rate	0.551

**Table 3:** Compound-Eye Apposition Concatenation Optimization Test Results.

	Unstitched Image		CEAC (2^2^)			CEAC (3^2^)			CEAC (4^2^)		
	mAP@50	Time	mAP@50	Time	Time (Average)	mAP@50	Time	Time (Average)	mAP@50	Time	Time (Average)
YOLOv13	92.19	16.4	91.97	16.8	4.2	91.74	17.9	2.0	88.15	18.6	1.2
+DC	92.92	16.2	92.43	16.5	4.1	92.12	17.4	1.9	88.47	18.3	1.1
+Gv2	92.84	15.6	92.28	15.8	4.0	91.93	16.8	1.9	88.28	17.4	1.1
+PST	92.37	15.0	92.11	15.5	3.9	91.84	16.6	1.8	88.22	17.1	1.1

Note: The time unit is unified as milliseconds. CEAC stands for Compound-Eye Apposition Concatenation. DC represents the DynamicConv improvement. Gv2 refers to the GhostNetv2 improvement. PST stands for Pyramid Sparse Transformer.

**Table 4:** MC-YOLOv13-L Network Parameters.

ID	From	Params	Module	Arguments
0	−1	464	Conv	[3, 16, 3, 2]
1	−1	2368	Conv	[16, 32, 3, 2, 1, 2]
2	−1	6424	C3k2_MSLA	[32, 64, 1, False, 0.25]
3	−1	9344	Conv	[64, 64, 3, 2, 1, 4]
4	−1	23,724	C3k2_MSLA	[64, 128, 1, False, 0.25]
5	−1	17,792	DSConv	[128, 128, 3, 2]
6	−1	174,720	A2C2f	[128, 128, 2, True, 4]
7	−1	34,432	DSConv	[128, 256, 3, 2]
8	−1	677,120	A2C2f	[256, 256, 2, True, 1]
9	−1	1,121,792	CMUNeXt	[256, 256, 1]
10	[4, 6, 8]	273,536	HyperACE	[128, 128, 1, 4, True, True, 0.5, 1, ‘both’]
11	−1	0	Upsample	[None, 2, ‘nearest’]
12	10	33,280	DownsampleConv	[128]
13	[6, 10]	1	FullPAD_Tunnel	[]
14	[4, 11]	1	FullPAD_Tunnel	[]
15	[9, 12]	1	FullPAD_Tunnel	[]
16	−1	0	Upsample	[None, 2, ‘nearest’]
17	[−1, 13]	0	Concat	[1]
18	−1	96,600	C3k2_MSLA	[384, 128, 1, True]
19	[−1, 10]	1	FullPAD_Tunnel	[]
20	18	0	Upsample	[None, 2, ‘nearest’]
21	[−1, 14]	0	Concat	[1]
22	−1	29,232	C3k2_MSLA	[256, 64, 1, True]
23	11	8320	Conv	[128, 64, 1, 1]
24	[22, 23]	1	FullPAD_Tunnel	[]
25	−1	36,992	Conv	[64, 64, 3, 2]
26	[−1, 19]	0	Concat	[1]
27	−1	72,024	C3k2_MSLA	[192, 128, 1, True]
28	[−1, 10]	1	FullPAD_Tunnel	[]
29	27	147,712	Conv	[128, 128, 3, 2]
30	[−1, 15]	0	Concat	[1]
31	−1	280,232	C3k2_MSLA	[384, 256, 1, True]
32	[−1, 12]	1	FullPAD_Tunnel	[]
33	[24, 28, 32]	431,452	Detect	[4, [64, 128, 256]]

**Table 5:** Hardware and Software Parameters.

Hardware/Software Name	Configuration Parameters
Operating System	Windows 10
Processor	12th Gen Intel(R) Core(TM)i5-12600KF
Graphics Card	NVIDIA GeForce RTX 4060 Ti (16 GB)
Solid-State Drive	Kingston NV2 1TB PCIe 4.0 NVMe M.2
Memory	Colorful 32(16×2) G 3200 DDR4
Driver	NVIDIA-SMI 561.09
CUDA	CUDA Version: 12.6
Programming Language	Python 3.9
Network Development	PyCharm 2024

**Table 6:** Parameter Settings.

Parameter	Value
Epochs	500
Batch	16
Input image size	640 × 640
Initial learning rate	0.1
Box loss gain	7.5
Classification loss gain	0.5
Distribution Focal Loss gain	1.5

**Table 7:** Ablation Study Results.

Model	Precision (%)	Recall (%)	mAP@50 (%)	Avg-mAP@50 (%)	FLOPs (G)	Parameters	mAP@50-95 (%)	FPS
YOLOv13	88.39	88.08	91.74	90.34 ± 0.78	6.4	2,460,691	72.98	55.87
M-YOLOv13	88.94	91.84	92.49	91.10 ± 1.09	6.2	2,355,775	74.27	58.14
C-YOLOv13	89.02	92.21	93.10	91.96 ± 0.82	7.3	3,582,483	74.39	54.64
YOLOv13-L	89.11	91.55	92.82	91.77 ± 0.67	6.4	2,460,691	74.34	57.14
MC-YOLOv13	89.67	92.32	94.02	93.15 ± 0.73	7.1	3,477,567	75.71	55.25
M-YOLOv13-L	91.01	91.49	93.78	92.71 ± 1.00	6.2	2,355,775	74.74	58.48
C-YOLOv13-L	92.15	94.78	94.24	93.69 ± 0.33	7.3	3,582,483	76.58	54.95
MC-YOLOv13-L	93.03	95.01	95.94	95.16 ± 0.53	7.1	3,477,567	78.05	55.24

Note: M: Multi-scale Linear Attention Mechanism Optimization; C: CMUNeXt Module Optimization; L: Loss Function Optimization; Avg: Average.

**Table 8:** Model Comparison Experiment Results.

Model	Precision (%)	Recall (%)	F1 (%)	mAP@50 (%)
MC-YOLOv13-L	93.03	95.01	94.01	95.94
YOLOv13	88.39	88.08	88.23	91.74
YOLOv10	86.63	87.29	86.96	90.65
SSD	75.37	80.28	77.75	81.35
RT-DETR	86.35	84.93	85.63	88.75
Faster-RCNN	75.24	76.02	75.63	78.62

## Data Availability

The original code presented in the study are openly available in IEEE DataPort at https://dx.doi.org/10.21227/v32y-mv49.

## References

[1] Zhao X., Wang H., Gao F., Wang P., Wang B. (2022). Realization of sustainable development goals in the tea industry: A case study of Lincang City, Yunnan Province. Adv. Earth Sci..

[2] Zhao Y., Zheng R., Zheng F., Zhong K., Fu J., Zhang J., Flanagan D.C., Xu X., Li Z. (2023). Spatiotemporal distribution of agrometeorological disasters in China and its impact on grain yield under climate change. Int. J. Disaster Risk Reduct..

[3] Hasan R., Islam A.F.M.S., Maleque M.A., Islam M.S., Rahman M.M. (2023). Effect of drought stress on leaf productivity and liquor quality of tea: A Review. Asian J. Soil Sci. Plant Nutr..

[4] Mirzaei S., Boloorani A.D., Bahrami H.A., Alavipanah S.K., Mousivand A., Mouazen A.M. (2022). Minimising the effect of moisture on soil property prediction accuracy using external parameter orthogonalization. Soil Tillage Res..

[5] Fatemi R., Yarnia M., Mohammadi S., Vand E.K., Mirashkari B. (2023). Screening barley genotypes in terms of some quantitative and qualitative characteristics under normal and water deficit stress conditions. Asian J. Agric. Biol..

[6] Driever S.M., Mossink L., Ocaña D.N., Kaiser E. (2023). A simple system for phenotyping of plant transpiration and stomatal conductance response to drought. Plant Sci..

[7] Mahdavi Z., Rashidi V., Yarnia M., Aharizad S., Roustaii M. (2023). Evaluation of yield traits and tolerance indices of different wheat genotypes under drought stress conditions. Cereal Res. Commun..

[8] Liang D., Zhou Q., Ling C., Gao L., Mu X., Liao Z. (2023). Research progress on the application of hyperspectral imaging techniques in tea science. J. Chemom..

[9] Ahmed R.M. (2024). Integration of wireless sensor networks, Internet of Things, artificial intelligence, and deep learning in smart agriculture: A comprehensive survey: Integration of wireless sensor networks, Internet of Things. J. Innov. Intell. Comput. Emerg. Technol. (JIICET).

[10] Márquez-Grajales A., Villegas-Vega R., Salas-Martínez F., Acosta-Mesa H.G., Mezura-Montes E. (2024). Characterizing drought prediction with deep learning: A literature review. MethodsX.

[11] Ali T., Rehman S.U., Ali S., Mahmood K., Aparicio Obregon S., Calderón Iglesias R., Khurshaid T., Ashraf I. (2024). Smart agriculture: Utilizing machine learning and deep learning for drought stress identification in crops. Sci. Rep..

[12] Zhou L., Zhang H., Bian L., Tian Y., Zhou H. (2024). Phenotyping of drought-stressed poplar saplings using exemplar-based data generation and leaf-level structural analysis. Plant Phenomics.

[13] Hu Y., Li Z., Lu Z., Jia X., Wang P., Liu X. (2024). Identification Method of Crop Aphids Based on Bionic Attention. Agronomy.

[14] Huang S., Lin C., Jiang X., Qu Z. (2024). Brstd: Bio-inspired remote sensing tiny object detection. IEEE Trans. Geosci. Remote Sens..

[15] Arief M.A.A., Kim H., Kurniawan H., Nugroho A.P., Kim T., Cho B.-K. (2023). Chlorophyll Fluorescence Imaging for Early Detection of Drought and Heat Stress in Strawberry Plants. Plants.

[16] Chen X., Shi D., Zhang H., Pérez J.A.S., Yang X., Li M. (2024). Early diagnosis of greenhouse cucumber downy mildew in seedling stage using chlorophyll fluorescence imaging technology. Biosyst. Eng..

[17] Liu S.B., Xie B.K., Yuan R.Y., Zhang M.X., Xu J.C., Li L., Wang Q.H. (2023). Deep learning enables parallel camera with enhanced-resolution and computational zoom imaging. PhotoniX.

[18] Shi J., Wang Y., Yu Z., Li G., Hong X., Wang F., Gong Y. (2023). Exploiting multi-scale parallel self-attention and local variation via dual-branch transformer-CNN structure for face super-resolution. IEEE Trans. Multimed..

[19] Cai H., Li J., Hu M., Gan C., Han S. (2022). Efficientvit: Multi-scale linear attention for high-resolution dense prediction. arXiv.

[20] Zhang Z., Xu Q., Shi H., Zhao G., Gao L., Wang T., Gu G., Jia L.Q. (2024). FSUNet: Lightweight full-scale information fusion UNet for seed coat thickness measurement. Cogent Food Agric..

[21] Yang Y., Wang Y., Zhu C., Xie Z., Qin Z., Wang Z., Chai Y. (2025). Bioinspired and biointegrated vision for artificial sight convergence. Nat. Rev. Bioeng..

[22] Zhang J., Zhou H., Wang S. (2024). Distinct visual processing networks for foveal and peripheral visual fields. Commun. Biol..

[23] Zhang H., Xu C., Zhang S. (2023). Inner-IoU: More effective intersection over union loss with auxiliary bounding box. arXiv.

[24] Maraveas C., Asteris P.G., Arvanitis K.G., Bartzanas T., Loukatos D. (2023). Application of bio and nature-inspired algorithms in agricultural engineering. Arch. Comput. Methods Eng..

[25] Yao J., Li M., Wu Z., Jiang C., An Y., Huang L., Chen N., Zhang J., Lu M. (2025). PagSAMDC4a-Mediated Polyamine Synthesis Regulate Vessel Differentiation Under Drought Stress Conditions in Poplar. Plant Biotechnol. J..

[26] (2015). Grade of Agricultural Drought.

[27] (2022). Tea Garden Drought Severity.

[28] Wu C., Wang D., Huang K. (2024). Enhancement of mine images based on hsv color space. IEEE Access.

[29] Devi T.G., Patil N., Rai S., Philipose C.S. (2023). Gaussian blurring technique for detecting and classifying acute lymphoblastic leukemia cancer cells from microscopic biopsy images. Life.

[30] Yuan W., Yang C., Wang X., Wang Q., Chen L., Zou M., Fan Z., Zhou M., Wang B. (2025). CV-YOLOv10-AR-M: Foreign Object Detection in Pu-Erh Tea Based on Five-Fold Cross-Validation. Foods.

[31] Lei M., Li S., Wu Y., Hu H., Zhou Y., Zheng X., Ding G., Du S., Wu Z., Gao Y. (2025). YOLOv13: Real-Time Object Detection with Hypergraph-Enhanced Adaptive Visual Perception. arXiv.

[32] He J., Wang W. (2025). NST−YOLO: Improved YOLOv10 model for small target UAV detection. Ain Shams Eng. J..

[33] Payawal J.M.G., Kim D.K. (2024). A review on the latest advancements and innovation trends in vibration-based structural health monitoring (SHM) techniques for improved maintenance of steel slit damper (SSD). IEEE Access.

[34] Zhao Z., Chen S., Ge Y., Yang P., Wang Y., Song Y. (2024). Rt-detr-tomato: Tomato target detection algorithm based on improved rt-detr for agricultural safety production. Appl. Sci..

[35] Sun X., Wu P., Hoi S.C. (2018). Face detection using deep learning: An improved faster RCNN approach. Neurocomputing.

[36] Srinivasu P.N., Kumari G.L.A., Narahari S.C., Ahmed S., Alhumam A. (2025). Exploring the impact of hyperparameter and data augmentation in YOLO V10 for accurate bone fracture detection from X-ray images. Sci. Rep..

[37] Liao L., Song C., Wu S., Fu J. (2025). A novel YOLOv10-based algorithm for accurate steel surface defect detection. Sensors.

[38] Quach L.D., Quoc K.N., Quynh A.N., Ngoc H.T., Thai-Nghe N. (2024). Tomato health monitoring system: Tomato classification, detection, and counting system based on YOLOv8 model with explainable MobileNet models using Grad-CAM++. IEEE Access.

